# Evaluation of linkage disequilibrium and its effect on non-parametric multipoint linkage analysis using two high density single-nucleotide polymorphism mapping panels

**DOI:** 10.1186/1471-2156-6-S1-S85

**Published:** 2005-12-30

**Authors:** Sarah Shaw Murray

**Affiliations:** 1 Illumina, Inc., 9885 Towne Centre Drive, San Diego, CA 92121, USA

## Abstract

Genotype data from the Illumina Linkage III SNP panel (*n *= 4,720 SNPs) and the Affymetrix 10 k mapping array (*n *= 11,120 SNPs) were used to test the effects of linkage disequilibrium (LD) between SNPs in a linkage analysis in the Collaborative Study on the Genetics of Alcoholism pedigree collection (143 pedigrees; 1,614 individuals). The average *r*^2 ^between adjacent markers across the genetic map was 0.099 ± 0.003 in the Illumina III panel and 0.17 ± 0.003 in the Affymetrix 10 k array. In order to determine the effect of LD between marker loci in a nonparametric multipoint linkage analysis, markers in strong LD with another marker (*r*^2 ^> 0.40) were removed (*n *= 471 loci in the Illumina panel; *n *= 1,804 loci in the Affymetrix panel) and the linkage analysis results were compared to the results using the entire marker sets. In all analyses using the ALDX1 phenotype, 8 linkage regions on 5 chromosomes (2, 7, 10, 11, X) were detected (peak markers *p *< 0.01), and the Illumina panel detected an additional region on chromosome 6. Analysis of the same pedigree set and ALDX1 phenotype using short tandem repeat markers (STRs) resulted in 3 linkage regions on 3 chromosomes (peak markers *p *< 0.01). These results suggest that in this pedigree set, LD between loci with spacing similar to the SNP panels tested may not significantly affect the overall detection of linkage regions in a genome scan. Moreover, since the data quality and information content are greatly improved in the SNP panels over STR genotyping methods, new linkage regions may be identified due to higher information content and data quality in a dense SNP linkage panel.

## Background

For many years short tandem repeat (STR) or microsatellite markers have been used as the standard genetic markers for linkage mapping [1-5]. STRs typically have a high degree of heterozygosity and provide high information content per marker [6]. They occur widely throughout the genome, with over 5,000 STRs being mapped to a high-resolution genetic map [5]. However, a drawback to using microsatellite markers is that because of the large number of alleles differing by as few as 2 to 4 bp, analysis has required electrophoretic separation. Because of this technical limitation, their analysis is time consuming and not amenable to highly multiplexed, automated formats.

In contrast, single-nucleotide polymorphism (SNP) assays are more amenable to multiplexing and are easier to automate, which increases accuracy and reliability. Therefore, more complete linkage studies with thousands of markers, and studies with large numbers of DNA samples are more feasible using SNPs. In addition, there is an abundance of SNPs: currently over 8 million human SNPs are in public databases, many of which have been validated . Recent reports have shown SNP linkage panels have higher information content and data quality than STR marker panels [7-9]. However, an important consideration for using numerous closely spaced SNPs in a linkage analysis is the analytical method used for statistical analysis of the genotype data. Genotyping many SNPs at high density compensates for the fact that each individual SNP has limited heterozygosity (maximum theoretical heterozygosity = 50%). Therefore, in order to maximize the information content (IC), one must use multipoint linkage analysis across a map of densely spaced SNPs or use multi-SNP haplotypes in a two-point linkage analysis. Another important consideration is the amount of linkage disequilibrium (LD) that exists between loci when using a mapping panel of densely spaced SNPs in a linkage analysis. Existing linkage analysis programs assume linkage equilibrium between loci. This assumption is violated if LD exists between SNP loci and may introduce a bias in the resulting LOD score. This report describes the results of determining the amount of LD that exists between loci in two different high-density SNP linkage panels and their effect on the overall linkage analysis results using 143 Collaborative Study on the Genetics of Alcoholism (COGA) pedigrees segregating the ALDX1 phenotype.

## Methods

### Samples

One hundred forty-three pedigrees from the COGA study comprised 1,614 individuals (1,332 genotyped individuals) segregating alcoholism were used in all analyses. The 143 pedigrees had 256 sibships. Of these 256 sibships, 123 (48%) had both parents, 75 (29%) had 1 parent, and 58 (23%) had no parents available for SNP genotyping. The majority of the individuals had a self-reported ethnicity of White, non-Hispanic (*n *= 1,074). The next largest self-reported ethnic group was black, non-Hispanic (*n *= 191).

### Genetic markers

The Illumina Linkage III SNP panel and the Affymetrix 10 k mapping array were used for both LD calculations and linkage analyses. The Illumina panel used for the Genetic Analysis Workshop 14 study had 4,720 SNP loci with a mean and median physical spacing of 615 kb and 406 kb, respectively. The data were of exceptional quality with a high call rate (99.95%), low error rate (0.005%), and low rate of Mendelian inconsistencies (0.09%). From a genetic map generated from 28 CEPH pedigrees [9], the mean and median genetic spacing was 1.5 cM and 1.1 cM, respectively. The Affymetrix 10 k mapping array had 11,120 loci with a mean and median physical spacing of 210 kb and 105 kb, respectively. The data were also of high quality with a 94.75% call rate, 0.052% error rate, and 0.12% rate of Mendelian inconsistencies. From a genetic map derived from linear interpolation of physical map position and a high resolution microsatellite genetic map, the mean genetic spacing was 0.32 cM [8]. The COGA STR marker set had 328 markers with an average genetic spacing of 10.45 cM. Previously the data quality for the COGA STR dataset were reported [10], and the missing genotype rate and Mendelian inconsistency rate were grouped together and estimated to be 4%. The error rate was estimated to be 0.8%.

### Evaluation of inter-marker LD

Inter-marker LD was determined from between all SNP loci in the two SNP panels, respectively, in 244 unrelated individuals. LD strength was derived from estimated haplotypes using the expectation-maximization algorithm of Slatkin and Excoffier [11] as implemented in the ldmax component of the program GOLD [12]. LD strength, as measured by the parameter *r*^2^, was determined for each pair-wise combination. In particular, LD between each adjacent marker on the genetic map used for linkage analysis was investigated. Loci in strong LD with another marker (*r*^2 ^> 0.40) were removed and the linkage analysis was repeated using the subset of markers where all loci have no or weak LD (*r*^2 ^< 0.40).

### Multipoint nonparametric linkage (NPL) analysis

Linkage analysis of both SNP panels and the COGA STR panel were analyzed using the ALDX1 phenotype. Genotype data were analyzed using multipoint nonparametric methods as implemented in GENEHUNTER v2.1 [13] on all pedigrees using the map distances provided with the genotype data. For the SNP loci, because the markers are densely mapped, multipoint analyses were carried out using one step between each marker. For the STR loci, multipoint analyses were carried out using four steps between each marker. Peak loci were determined as those regions with *p*-values < 0.01.

## Results

### Inter-marker LD

**Figure 1:**
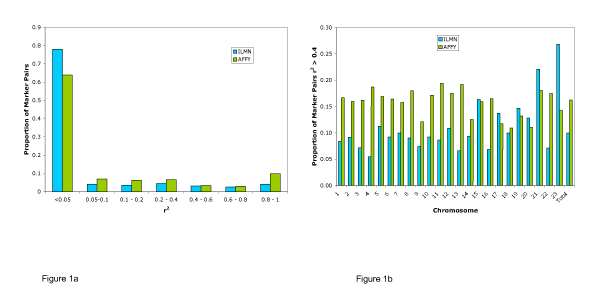
**LD strength in the Illumina Linkage III SNP panel (ILMN) and the Affymetrix 10 k mapping array (AFFY). **a) Distribution of marker pairs by LD strength (*r*^2^); b) Proportion of marker pairs *r*^2 ^> 0.4 by chromosome (chromosome 23 = X chromosome).

LD strength (*r*^2^) between each adjacent marker pair on the genetic map was evaluated in both SNP panels. For the Illumina SNP panel, the average *r*^2 ^between each adjacent marker pair on the genetic map was 0.099 ± 0.003 (SD = 0.23). As shown in Figure 1a, >75% of all marker pairs had an *r*^2 ^< 0.05. Figure 1b shows the proportion of marker pairs that have *r*^2 ^> 0.4 by chromosome. The X chromosome had the highest proportion of marker pairs with *r*^2 ^> 0.4 (33/123 marker pairs; 27%). Overall, 471/4,697 marker pairs (10%) had *r*^2 ^> 0.4. One marker from each of the 471 marker pairs with *r*^2 ^> 0.40 was randomly removed. In the case where there were multiple adjacent SNPs with *r*^2 ^> 0.40, the minimum numbers of loci were removed so that the remaining adjacent loci had *r*^2 ^< 0.40. A subset of 4,149 loci was used for subsequent linkage analyses where all loci had weak or no LD. For the Affymetrix SNP panel, the average LD between loci was higher (as expected) because the SNPs are more closely spaced. The average *r*^2 ^between each adjacent marker pair on the genetic map was 0.17 ± 0.003 (SD = 0.30), and less than 65% of all marker pairs had an *r*^2 ^< 0.05 (Figure 1a). There were 1,804 marker pairs with *r*^2 ^> 0.40. One marker from each pair was removed for subsequent linkage analysis using the remaining 9,316 loci with weak or no LD.

### NPL multipoint analysis

**Figure 2:**
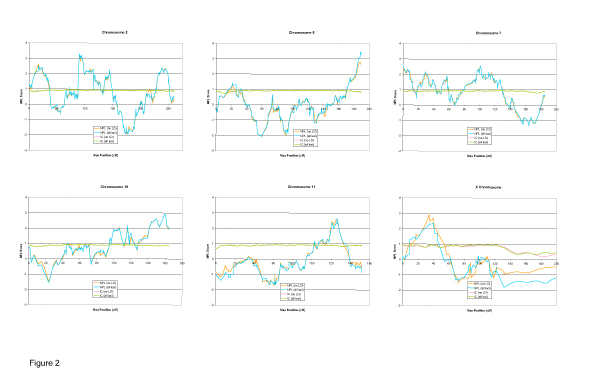
**Multipoint NPL analysis of ALDX1 phenotype using the Illumina III SNP linkage panel. **Multipoint NPL scores across the entire chromosome for all SNPs (blue line) and a subset of SNPs with weak or no LD (orange line) are shown for the six chromosomes that have regions with corresponding p-values < 0.01. In addition, the information content (IC) curves are also shown for all loci (green line) and the subset of SNPs with weak or no LD (pink line).

**Figure 3:**
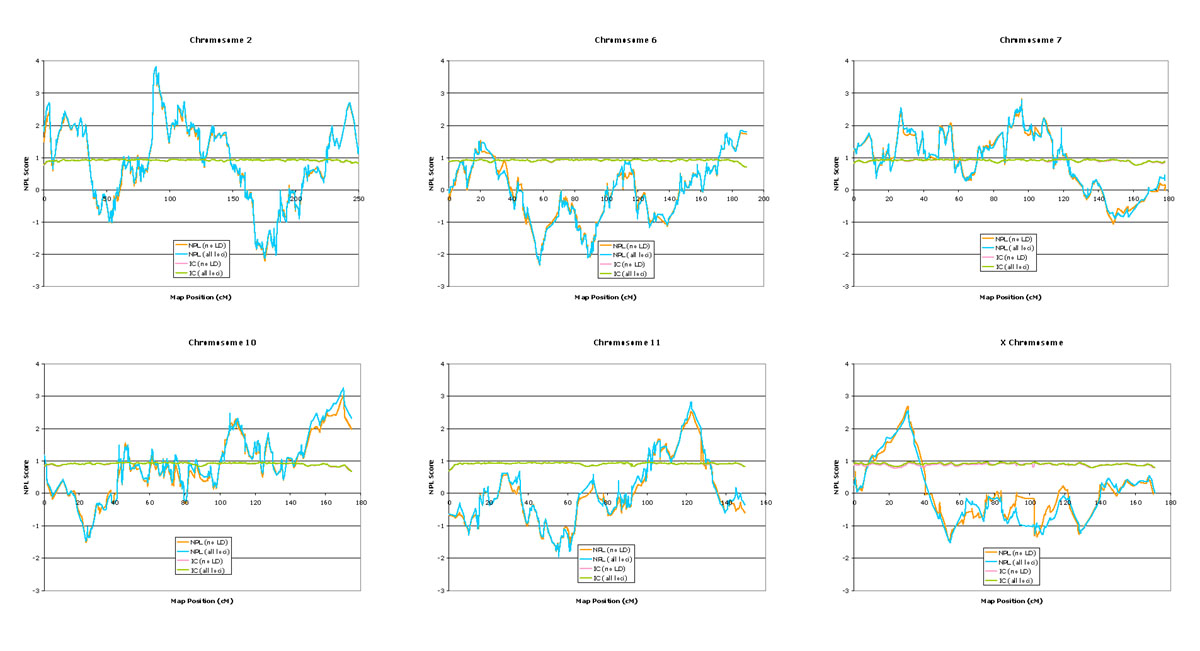
**Multipoint NPL analysis of ALDX1 phenotype using the Affymetrix 10 k Mapping Array**. Multipoint NPL scores across the entire chromosome for all SNPs (blue line) and a subset of SNPs with weak or no LD (orange line) are shown for six chromosomes. Chromosomes 2, 7, 10, 11, and X have regions with corresponding *p*-values < 0.01; chromosome 6 is shown for comparison with the Illumina SNP mapping panel results. In addition, the information content (IC) curves are also shown for all loci (green line) and the subset of SNPs with weak or no LD (pink line).

Genotype data were analyzed using the ALDX1 phenotype and the NPL multipoint analysis methods as implemented in the program GENEHUNTER v2.1 [12]. For the complete set of 4,720 SNP loci in the Illumina SNP panel, nine regions on six chromosomes (chromosomes 2, 6, 7, 10, 11, X) had peak NPL scores with corresponding *p*-values < 0.01 and are summarized in Table 1. Next, a subset of the Illumina SNP panel was analyzed where all 4,149 loci had weak or no LD (*r*^2 ^< 0.40). The same nine regions on six chromosomes were identified in this analysis. These results are also shown in Table 1. The biggest difference in results between the two analyses (all loci versus loci with no or weak LD) occurred on the X chromosome, where a high proportion of loci (17%) were removed and the IC at the linkage peak dropped from 0.93 to 0.88. The linkage peak shifted approximately 5 cM between the two analyses. Another big difference between the two analyses occurred on chromosome 6, where 3 of the 9 loci in the interval were removed, including the peak marker from the analysis of all loci. The analysis using a subset of markers was still significant (*p *= 0.004), though less significant than the analysis with all loci (*p *= 0.0006). Even though there were moderate differences between the two analyses on these two chromosomes, the same regions were identified in both analyses with *p*-values ≤ 0.01. The six chromosomes with *p*-values ≤ 0.01 are shown in Figure 2. For the complete set of 11,120 loci in the Affymetrix 10 k Mapping Array, eight regions on five chromosomes (chromosomes 2, 7, 10, 11, X) had peak NPL scores with corresponding *p*-values < 0.01 as shown in Table 1. A second analysis without the 1,804 loci in strong LD resulted in the same eight regions and similar NPL scores and *p*-values (Table 1). The Affymetrix SNP panel did detect a peak at the same region on chromosome 6 as in the Illumina SNP panel, however the *p*-value was > 0.01 (Table 1, Figure 3). The five chromosomes with linkage regions (*p *< 0.01) are shown in Figure 3.

IC was also compared across all marker sets as shown in Table 2. The genome-wide average IC for the Illumina set was 0.89 (min = 0.33, max = 0.97) with only one chromosome having an average IC < 0.80. In addition, the IC was consistently high across most of the genome (with the exception of a gap in Xq23-q28), with 2 chromosomes having a minimum IC less than 0.70. The genome-wide average IC of the Affymetrix set was slightly higher (average IC = 0.90, range = 0.43–0.97) with all chromosomes having an average IC > 0.80. Even though this panel had over twice the number of loci, there were only modest improvements in average IC, and actually there were more regions of lower IC, since 8 chromosomes had a minimum IC < 0.70.

Finally, STR data were analyzed using the ALDX1 phenotype. Two of the 9 linkage peaks detected using the high-density SNP map were detected with STRs at *p *< 0.01. The resulting peak loci and linkage intervals are summarized in Table 3. The peak on chromosome 7 does not overlap with the two peaks on chromosome 7 detected using high density SNPs, however it is flanked by the two peaks detected in the SNP panels. As shown in Table 2, the genome-wide IC was considerably lower than both SNP panels (average = 0.61), since no chromosomes had an average IC > 0.80. In addition, all chromosomes had minimum IC < 0.70.

## Discussion

An early study suggested that if LD exists between a trait and marker and if LD is not taken into account in a linkage analysis, the resulting LOD score would be reduced [14]. However, LD that exists between two marker loci may not necessarily have the same bias, and could in fact artificially increase a LOD score in a linkage analysis. For example, if haplotype frequencies are calculated assuming linkage equilibrium when in fact LD exists and the linked haplotype frequency is underestimated, then this could artificially inflate the resulting LOD score [15]. This effect would be largest in pedigrees in which genotypes are not available for founding individuals. In this study using the Illumina SNP panel, a low level of LD was detected between each SNP overall, and 78% of all marker pairs in order of the genetic map had an *r*^2 ^< 0.05. Using the Affymetrix mapping array, a higher proportion of marker pairs had high LD genome-wide, and 65% of all marker pairs had an *r*^2 ^< 0.05.

Overall, in both SNP panels there was very little difference between results using the entire marker set and the reduced marker set without loci in strong LD. Using both SNP panels, the same regions were detected in both analyses at *p *< 0.01, with modest changes in *p*-values and NPL scores. The NPL scores and *p*-values did not consistently become more or less significant with the removal of loci in LD. In addition, there was high concordance in terms of significance and location of linkage peaks between the two SNP mapping panels. The main difference in findings between the two panels was that the Affymetrix mapping array did not detect the linkage peak at *p *< 0.01 on chromosome 6. This result could have occurred for many reasons. The IC is lower in this region (IC = 0.81 at peak marker). In addition, because the peak marker does not map uniquely in the build 34 genome assembly, the genetic map position may not be accurate since the genetic map position is linked to physical map position through linear interpolation in this SNP panel. Finally, this result could be a false-positive result in the Illumina mapping panel.

The analysis of STR markers in the same pedigree set resulted in only 3 linkage peaks with corresponding *p*-values < 0.01. Two of these regions (2p14, 11q23) overlapped with the SNP linkage results and the third region (7p14) did not overlap although it was between two of the SNP linkage peaks (7p22, 7q21). In addition, the IC using the STR data was approximately 3–21% lower at the linkage peaks compared with the high density SNP data.

## Conclusion

The results on this study suggest that LD between loci in a linkage analysis does not significantly affect the overall detection of linkage regions in a genome scan. However, this result is dependent on the number of genotyped founders or unaffected siblings in the pedigrees because the potential bias in LOD score due to underlying LD between SNP loci in a linkage analysis is largest in pedigrees in which genotypes are not available for founding individuals. Therefore, one strategy to determine the effect that LD has on a linkage analysis in other pedigree collections might be to re-run a linkage analysis using a subset of loci with weak or no LD in the pedigree collection being studied. Therefore, one can determine if a linkage result is being inflated by underlying LD between SNP loci in the linkage peak. Despite the potential bias of a resulting LOD score, the high-density SNP panels provide an order of magnitude higher data quality compared to STR genotyping methods and also provide higher IC. In this study, several new linkage regions may have been identified that were not detected using a 10-cM STR marker panel due to higher IC and data quality in the dense SNP linkage panels.

## Abbreviations

COGA: Collaborative Study of the Genetic of Acoholism

IC: Information Content

LD: Linkage disequilibrium

NPL: Nonparametric linkage

STR: Short tandem repeat marker

SNP: Single-nucleotide polymorphism

## Figures and Tables

**Table 1:** Linkage results using the Illumina Linkage III SNP Panel and the Affymetrix 10 k Mapping Array.

	Illumina III SNP linkage panel						Affymetrix 10 k mapping array					
		
Chr	Marker	Position (cM)	Build34 bp	NPL	*p*-value	IC	Marker	Position (cM)	Build34 bp	NPL	*p*-value	IC
**2p25**	**rs1025053**	**16.1**	**8238143**	**2.53**	**0.0073**	**0.89**	**tsc0041232**	**5.74**	**2888794**	**2.71**	**0.0047**	**0.91**
2p25	rs1025053	16.1	8238143	2.64	0.0056	0.90	tsc0041232	5.74	2888794	2.43	0.0095	0.91
**2p14**	**rs1000756**	**89.9**	**66769368**	**3.31**	**0.00090**	**0.93**	**tsc1090282**	**90.58**	**67905612**	**3.82**	**0.00019**	**0.94**
2p14	rs1106577	89.9	67023129	3.26	0.0010	0.93	tsc1090282	90.58	67905612	3.80	0.00021	0.94
**2q37**	**rs1453867**	**239.2**	**233120438**	**2.39**	**0.010**	**0.93**	**tsc0524987**	**244.44**	**236010878**	**2.71**	**0.0046**	**0.89**
2q37	rs1453867	239.2	233120438	2.38	0.010	0.93	tsc0524987	244.44	236010878	2.68	0.0051	0.89
**6q27**	**rs760909**	**188.1**	**170713389**	**3.46**	**0.00058**	**0.86**	**tsc0050465**	**185.42**	**multiple pos.**	**1.85**	**0.035**	**0.81**
6q27	rs727619	188.1	170562703	2.79	0.0037	0.88	tsc0050465	185.42	multiple pos.	1.77	0.041	0.81
**7p22-21**	**rs1057558**	**0**	**806704**	**2.62**	**0.0058**	**0.89**	**tsc1592599**	**31.68**	**17610703**	**2.55**	**0.0070**	**0.94**
7p22-21	rs1127460	0	623105	2.35	0.011	0.89	tsc0925001	31.35	17305065	2.42	0.0096	0.93
**7q21**	**rs1023564**	**101.1**	**88453743**	**2.54**	**0.0071**	**0.94**	**tsc0049271**	**101.05**	**89624115**	**2.77**	**0.0039**	**0.94**
7q21	rs717474	101.1	88403326	2.54	0.0072	0.94	tsc1240889	99.05	85964828	2.82	0.0035	0.94
**10q26**	**rs1536087**	**159.6**	**131394560**	**2.90**	**0.0028**	**0.90**	**tsc0960887**	**171.41**	**132054567**	**3.25**	**0.0011**	**0.88**
10q26	rs1536087	159.6	131394560	2.92	0.0027	0.90	tsc0960887	171.41	132054567	3.03	0.0020	0.87
**11q23**	**rs617847**	**126.1**	**120940295**	**2.65**	**0.0054**	**0.90**	**tsc0053512**	**125.27**	**121196164**	**2.82**	**0.0035**	**0.92**
11q23	rs575030	125.1	120630050	2.57	0.0066	0.91	tsc0051264	125.15	121112747	2.51	0.0076	0.92
**Xp22**	**rs225031**	**39.7**	**28452585**	**2.34**	**0.011**	**0.93**	**tsc0038428**	**42.78**	**25961925**	**2.55**	**0.0071**	**0.97**
Xp22	rs795489	34.4	23062119	2.89	0.0030	0.88	tsc0038428	42.78	25961925	2.68	0.0053	0.94

^a^Peak loci *p *< 0.01 for all loci (bold) and loci *r*^2 ^< 0.40 (not bold)^1^

**Table 2:** Information content comparisons in the two SNP marker sets and the COGA STR marker set (Average (min, max))

Chromosome	Illumina		Affymetrix		COGA STR (all loci)
			
	all loci	*r*^2 ^< 0.4	all loci	*r*^2 ^< 0.4	
1	0.91 (0.75,0.95)	0.91 (0.75,0.96)	0.91 (0.69,0.95)	0.91 (0.69,0.95)	0.64 (0.28,0.90)
2	0.91 (0.79,0.95)	0.91 (0.79,0.95)	0.92 (0.78,0.96)	0.92 (0.77,0.96)	0.70 (0.47,0.90)
3	0.91 (0.82,0.95)	0.91 (0.83,0.95)	0.92 (0.84,0.97)	0.92 (0.83,0.97)	0.59 (0.41,0.86)
4	0.89 (0.72,0.96)	0.89 (0.72,0.95)	0.91 (0.59,0.96)	0.91 (0.59,0.96)	0.62 (0.47,0.78)
5	0.90 (0.77,0.95)	0.90 (0.77,0.95)	0.92 (0.75,0.96)	0.92 (0.75,0.96)	0.63 (0.37,0.85)
6	0.90 (0.72,0.96)	0.90 (0.71,0.95)	0.92 (0.71,0.96)	0.92 (0.71,0.96)	0.60 (0.37,0.84)
7	0.91 (0.78,0.95)	0.91 (0.78,0.95)	0.91 (0.78,0.96)	0.91 (0.77,0.95)	0.78 (0.59,0.92)
8	0.90 (0.79,0.95)	0.89 (0.75,0.95)	0.91 (0.75,0.95)	0.91 (0.75,0.95)	0.67 (0.44,0.87)
9	0.89 (0.78,0.95)	0.89 (0.77,0.95)	0.91 (0.70,0.96)	0.91 (0.69,0.96)	0.62 (0.47,0.85)
10	0.89 (0.78,0.95)	0.89 (0.78,0.95)	0.91 (0.68,0.95)	0.91 (0.68,0.95)	0.48 (0.31,0.76)
11	0.89 (0.66,0.95)	0.89 (0.66,0.95)	0.92 (0.70,0.96)	0.92 (0.69,0.96)	0.51 (0.30,0.74)
12	0.90 (0.75,0.95)	0.89 (0.75,0.95)	0.90 (0.74,0.96)	0.90 (0.74,0.96)	0.65 (0.38,0.90)
13	0.90 (0.77,0.95)	0.89 (0.76,0.95)	0.91 (0.62,0.96)	0.91 (0.62,0.96)	0.62 (0.33,0.86)
14	0.90 (0.79,0.95)	0.89 (0.79,0.95)	0.86 (0.43,0.95)	0.86 (0.42,0.95)	0.62 (0.49,0.79)
15	0.89 (0.77,0.95)	0.89 (0.76,0.95)	0.90 (0.83,0.95)	0.90 (0.83,0.95)	0.67 (0.56,0.85)
16	0.90 (0.75,0.95)	0.89 (0.74,0.95)	0.87 (0.46,0.95)	0.86 (0.46,0.95)	0.57 (0.33,0.82)
17	0.89 (0.79,0.94)	0.88 (0.78,0.94)	0.85 (0.51,0.94)	0.85 (0.51,0.94)	0.56 (0.35,0.77)
18	0.88 (0.75,0.95)	0.88 (0.75,0.95)	0.91 (0.70,0.95)	0.91 (0.70,0.95)	0.51 (0.35,0.69)
19	0.88 (0.73,0.95)	0.86 (0.71,0.94)	0.81 (0.57,0.94)	0.81 (0.56,0.94)	0.56 (0.41,0.76)
20	0.89 (0.82,0.94)	0.89 (0.80,0.94)	0.89 (0.78,0.95)	0.89 (0.71,0.95)	0.60 (0.39,0.82)
21	0.91 (0.85,0.94)	0.90 (0.84,0.94)	0.92 (0.75,0.95)	0.91 (0.75,0.95)	0.59 (0.39,0.83)
22	0.89 (0.79,0.95)	0.88 (0.78,0.95)	0.84 (0.77,0.94)	0.84 (0.77,0.94)	0.55 (0.34,0.76)
X	0.74 (0.33,0.97)	0.72 (0.20,0.97)	0.91 (0.84,0.97)	0.88 (0.78,0.95)	0.32 (0.24,0.40)
Genome	0.89 (0.33,0.97)	0.89 (0.20,0.96)	0.90 (0.43,0.97)	0.90 (0.42,0.97)	0.61 (0.24,0.92)
No. chr w/avg IC < 0.8	1	1	0	0	23
No. chr w/min IC < 0.7	2	2	8	10	23

**Table 3:** Linkage results using the COGA STR marker set (regions with peak *p*-values < 0.01)

Chromosome	Marker	Position (cM)	Build34 bp	NPL	LOD	*p*-value	IC
2p14	D2S285	94.2	67620904	2.56	1.42	0.0070	0.90
7p14	D7S2846	56.8	37875515	2.67	1.55	0.0052	0.86
11q23	D11S1998	119.8	117235383	2.61	1.47	0.0062	0.69
